# Supplementary material for: How Were Return-of-Service Schemes Developed and Implemented in Botswana, Eswatini and Lesotho?
Source: Healthcare (Basel). 2023 May 22;11(10):1512. doi: 10.3390/healthcare11101512 (PMC10218024; doi:10.3390/healthcare11101512)
Supplement: Supplementary file 1 [file healthcare-11-01512-s001.zip › healthcare-2387666-supplementary.pdf]

Supplementary S1

Data Extraction: Policy Review

A. General Information and Eligibility

1. Date form completed		
2. Name of person extracting data		
3. Report title		
4. Publication type		
5. Type of document		
6. Publication reference		
7. Country of publication		
8. Province		
9. Policy description		
10. Decision	Include	
	Exclude	
11. Notes (include reasons for exclusion):		

*No continuation if excluded.

B. Context

**Contextual variable**	**Description**	**Location in document**
12. Date of publication		
13. Version number		
14. Prior version Review Date		
15. Current version review Date		
16. Policy motivation and rationale		
17. Policy history		
18. Acts which informed policy		
19. Target beneficiaries		
20. Any other contextual issues		

C. Policy Content

Factor	Description	Location in document
21. Classification of beneficiaries		
22. Beneficiary selection criteria		
23. Policy objectives and/or purpose		
24. Skills-mix of beneficiaries		
25. Conditions of the funding		
26. Duration of funding		
27. Budgetary implications		
28. Administration of scheme		
29. Beneficiary responsibilities		
30. Person/body responsible for admitting beneficiaries into scheme.		
31. Any other factors:		

D. Process Implementation

Process	Description	Location in document
32. Statutory conditions for validity of policy/contract		
33. Term of policy		
34. Trigger for review of policy		
35. Trigger for evaluation of policy		
36. Skills or service needs determination process		
37. Beneficiary selection process		
38. Contract renewal process		
39. Beneficiary monitoring processes		

40. Placement of beneficiaries into services after completion of studies		
41. Any other processes		

E. Actors Involved

**Actors**	**Description**	**Location in document**

F. Conclusion

**Remark**	**Description**	**Location in document**

Supplementary S2: Interview Guide - Policymakers

Individual or Group Interview sheet and interview guide for return-of-service scheme policymakers and/or policy-implementers

Thank you so much for agreeing to take time from your busy schedule to answer a few questions on my research. I am doing a research study to find out more about the government policies used to fund health professionals in-training, in exchange for a period of service in the public health sector. The study aims to understand more about the history and evolution of these policies, how they relate with other human resources for health policies, their rationale, and how they are monitored and reviewed.

You are being interviewed because you are a manager that is involved in some way with the development and/or administration of policies that inform government sponsored bursaries or scholarships for health sciences students studying in the country or in other countries.

A group interview allows for a detailed discussion with a diverse group from the different units and divisions at once instead of hosting multitude of interviews with individuals within the department. That is the main reason why I have asked you to participate in the group interview. (For those who are unable to participate in a group interview this will read: I understand that it wasn't possible for you to be part of a group interview due to your schedule. Because I value your contribution it is for this reason that I still requested to have an individual discussion with you). Please do not be intimidated by anyone as the information collected will only be used to enrich the schemes. All the names from this discussion will be de-identified and your identified responses will not be shared outside the research team. Your individual and diverse inputs are therefore highly valued. The aim of the research is not to assess professional competence and the outcomes of the research will not have a negative impact on your employment. In addition, you are welcome to refer to internal human resources and/or bursary/scholarship scheme related documents or even consult colleagues who you think might help remind you of detail that you might have forgotten. It's also ok to not have all the answers. Please remember that the session is being audio recorded. You are welcome to let me know if you are not comfortable with that.

If you are happy with contents of this document and agree with the process could you kindly sign the consent forms and return to me before we start, if you haven't already done so. I am happy to answer any questions that you may have before we begin the discussion. Are there any questions that any of you would like to ask on the process before we start?

Date of administration: **Ethics approval number: HC200519**

No.	Area of Interest/topic	Initial broad descriptive questions	Possible probing questions
1	Origins and evolution of the policy	- What is the departmental policy on bursaries for health sciences students?<br>- What are the policy objectives?<br>- In your understanding and knowledge, what has influenced the bursary policy for health sciences students?<br>- As far as you know, when was this policy first introduced?<br>- Could you enlighten me more about the development process and implementation of the bursary policy?<br>- Which countries do beneficiaries of the policy go to for their studies?	- Could you please tell me more about any policy development frameworks used for developing your bursary policy or any other human resources for health policies?
2	Custodian of the policy	- Could you let me know which department or departments is or are responsible for the development and implementation of the bursary policy?<br>- Could you give more information about the role of any other departments, offices or sections that could be involved?<br>- Who makes the final decision on who receives an offer?	- How long has the situation been that way?<br>- How has the process evolved over time
3	Review of the policy	- Is the bursary policy regularly reviewed?<br>- What informs the reviewing of this policy?	- Is this related to political term?<br>- What informs the need to review this policy?
4	Decision process	- Could you tell me more about the process that informs the number of beneficiaries that can be funded in any particular funding cycle?<br>- How are the opportunities advertised?	- How do you decide between the various categories of health sciences students that you fund?

		- Can you tell me more about the selection criteria used to then select beneficiaries?	
5	Contract	- In your view, what are the responsibilities of bursary recipients?<br>- At what stage of the bursary offer do beneficiaries sign their contract?<br>- What are the key contents of the contract?<br>- What happens if a beneficiary defaults their contract?	- Is there an opt-out clause to the scheme? Elaborate…<br>- What happens if a beneficiary doesn't complete their studies?
6	Process after the completion of studies	- How are the new graduates recruited into the health system?<br>- At what stage of the process do you decide on the facility where the recipient will be placed in the health system?<br>- What processes are used to identify the types of facilities that need the placement of beneficiaries?<br>- At what stage do you plan for the salaries of beneficiaries?	- Are beneficiaries placed based on their own choices or on facilities chosen by government?<br>- Who decides on the placement of graduates who previously benefited from the bursary scheme?
7	Policy Challenges	- What challenges has the policy encountered over the years?	- To your knowledge, have recipients defaulted their contracts previously?<br>- What could be the reasons for beneficiaries to default bursary contracts?<br>- What is the sustainability of the policy?
8	Monitoring and evaluation of the policy	- What processes are in place to ensure that beneficiaries fulfil their contractual obligations?<br>- How often or uniformly are penalties imposed on those who default their contracts?	- What Information systems are in place to monitor fulfilment of the policy?

		- What features help or hinder monitoring of the program?<br>- In your view, does the policy fulfil its objective?<br>- If there is anything that you could change in the policy what would it be?<br>- In your view, what are the ways that could have helped eliminate defaulting of the scheme?	

Wrap-up

Are the any other issues not covered that you would like to talk about?

Thank you once more for your assistance, could you please also help me with a few documents that will help broaden my understanding on the schemes. You could add any other documents to the list if you think it or they will be of importance.

**List of documents to be requested**
1. All versions of bursary policy (current and historical) that are used to fund skilled health professionals.
2. Blank copies of bursary contracts.
3. Total health budget for the period 2000 to 2020.
4. Health sciences bursary budget for the period 2000 to 2020.
5. The total number of skilled health professionals (stratified by category and health facility) on 01 July 2020.
6. The total number of skilled health professionals who are bursary beneficiaries (stratified by category and health facility) employed in the contracted government service area on 01 July 2020.
7. Annual performance plans for the period 01 April 2015 to 31 March 2020.
8. Annual performance reports for the period 01 April 2016 to 31 March 2021.

Codebook

Name	Files	References
Aim of programme	6	22
Programme description	5	29
Application process	2	2
Advertisement	3	5
Barriers to policy implementation	2	2
Challenges	5	27
Aspirations hindered by lack of funds	1	3
Dynamism of the health system	1	2
Failure to employ beneficiaries	1	3
Feeling entitled	1	1
Relevance of training for country of origin	1	3
Unintended consequences	3	7
Training professionals for other countries	2	8
Beneficiary characteristics	6	37
Historical funding or debt	1	1

Name	Files	References
Previous discriminatory practices	1	1
Beneficiary obligations	6	44
Loan repayment terms	4	15
How are funds recovered	5	10
Benefits to individual	2	3
Benefits to system	1	1
Contents of Policy document	2	4
Contents of the contract	6	15
Contract Opt-out clause	2	4
Contract variation	4	8
Contracting process	1	1
Power differential	1	1
Timing of contract signing	2	3
Coordination between departments	5	20
Intersectoral collaboration	3	6
Countries of study	6	33
Academic institutions of study	3	5
International relations and source of funding	4	9

Name	Files	References
Critical skills shortages	2	2
Defaulting of schemes	6	35
How can schemes be strengthened to eliminate defaulting	1	1
Processes in place to track defaulters	4	11
Sanctions for breach	4	8
Employment guaranteed	1	3
Employment not guaranteed	3	18
Good practice	1	3
Governance of programme	2	7
Aspirations of the programme	1	1
Manager's experience	2	5
Organisational structure	2	4
Policy custodians	2	5
Recognition of inter-country Regional agreements	1	4
Weakness in Leadership	1	3
Government responsibilities	2	5
Government to Government agreements	1	2
Grading of Beneficiaries	2	8

Name	Files	References
How are beneficiaries recruited into the system	2	3
How are Beneficiaries selected	4	29
Advertisement	3	4
Transparency and oppenness of process	1	1
How are opportunities advertised	4	7
How much fees cost	1	1
How Schemes Work in Practice	5	15
Cost of the schemes	2	5
Incompetence	1	3
Innovation of building internal training capacity	2	2
Insufficient resources	2	2
Internal Programme Capacity	2	3
Lack of job availability	2	3
Lessons to learn	1	2
Loopholes	5	35
Monitoring during their studies	2	2
Monitoring post education	4	14
Information systems	4	17

Name	Files	References
Information availability and archiving	5	12
Interoperable Information System	3	14
Needs determination	6	28
Needs of country	3	7
New Node	0	0
Programme policy is looking to overcome	1	1
Number of beneficiaries	4	11
Number of potential beneficiaries	4	7
Number of times contract is signed	2	4
Opportunities	2	4
Commodifying health professionals who cannot be placed in employment	1	5
Origins of the scheme	6	17
Policy development framework	3	3
Placement into employment	5	18
Planning and budgeting	5	21
Driver of planning and skills needs determination	2	7

Name	Files	References
Plans to review policy	5	11
Reasons for need to review policy (outdated)	3	11
Policies should allow for some flexibility	1	5
Policy document	3	7
Policy Evaluation	3	3
Policy evolution	1	3
Poor academic performance	4	9
Possible solutions	2	3
Professional Registration post qualification	1	1
Programme exclusion criteria	1	3
Scheme configuration	0	0
Academic programmes	6	37
Scheme types	4	17
Social Responsibility	3	7
Need to address equity	2	2
Sustainability	5	14
Universality of skills	1	1
What is covered by funding	3	7

Checklist for the use and reporting of document analysis (CARDA)

Section and topic	Checklist item	Response/Action
Title	Identify the study involved in document analysis.	Done
Abstract	Identify the methods as mixed (listing the methods) or solely document analysis.	Done
Rationale	Describe the rationale for the use of documents in the study.	Done
Objectives	Provide an explicit statement of the research objective(s) or question(s) of the study.	Done
Eligibility criteria	Specify the eligibility criteria for including documents as data in this specific study.	Done
Document corpus		
Specify the nature of the documents in the corpus:	How many documents there were.	Done
	What kinds of documents were involved (e.g., local curriculum guides and national policy papers	Done
	The documents' media (print, electronic etc.).	Done
	The original purposes of the document where existing documents were used (e.g., Target audience. Who produced them, when and why?)	Done
	Include a table or equivalent documentation of all the documents used (either in the paper or as a supplementary table).	Done
Document provenance	State whether the documents were study-specific or elicited (created as part of data collection, e.g., field notes and diary entries) or existing (extant) documents (e.g., meeting minutes, prospectuses, policy or historical documents).	Done
Where documents were study-specific, specify:	How they were elicited and from whom.	Done
	Whether they were researcher- or participant-created. For example, where diary entries or reflective writing are used and explain why the rationale behind the target group of participants and the guidance given to participants in respect of producing texts.	N/A
	When the documents were created and when they were collected as part of the study (e.g., January 2020 to the end of December 2020).	Done
Where existing documents were used, specify:	How the documents were identified (e.g., archives or websites searched).	Done
	If appropriate, present the full search strategies for document identification, including any filters and limits used (e.g., English language only and particular websites only).	Done
	The data limits of any searches and the rationale for these data limits.	N/A
Document collection and management	How documents were obtained, managed etc.	Done
	Report if any of the documents used are publicly available and where they can be found.	Done

Document quality	Consider the "quality" of the documents and the relation of document quality to the study objectives.	Done
For existing documents:	Were they complete?	Done
	Were there gaps in the documents? Were they redacted?	Done
	Did you need to do more searching or rely on additional documents than planned?	Done
	Were some documents not available or accessible?	Done
For elicited documents:	Did participants engage in the process as intended?	Done
	Were the data comprehensive or sparse?	Done
	How much researcher effort was required to elicit the data, and what might be the implications of researcher interventions (e.g., frequent reminders)?	Done
Reflexivity/positionality (may be placed in the methods or discussion section of your paper)	Role and experience of researchers, experience in DA and positionality.	Done but separated from main document to comply with journal guidelines.
	Consider the potential presence of positionalities, both in a document and of the researcher(s)	Done
Preliminary data analysis	Specify the approach to preliminary or organising data analysis, the methods used to collect data from documents; often using variations on thematic or content analyses.	Done
	Specify if any automation tools were used in the process (e.g., AntConc and Wordsmith).	Done
Document analysis		
Outline the analytical steps taken—what methodology or methods were involved?	Explain whether the analysis focused on content, latent content, linguistics or some other document content or characteristics.	Done
	To what extent and in what ways did analysts immerse or attune themselves to the content, style, subtexts and other dimensions of the documents they analysed?	Done
	Was a theoretical lens used to ensure transferability?	

COREQ (COnsolidated criteria for REporting Qualitative research) Checklist

A checklist of items that should be included in reports of qualitative research. You must report the page number in your manuscript where you consider each of the items listed in this checklist. If you have not included this information, either revise your manuscript accordingly before submitting or note N/A.

Topic	Item No.	Guide Questions/Description	Reported on Page No.
**Domain 1: Research team and reflexivity**			
Personal characteristics			
Interviewer/facilitator	1	Which author/s conducted the interview or focus group?	5
Credentials	2	What were the researcher's credentials? E.g. PhD, MD	5
Occupation	3	What was their occupation at the time of the study?	5
Gender	4	Was the researcher male or female?	5
Experience and training	5	What experience or training did the researcher have?	5
Relationship with participants			
Relationship established	6	Was a relationship established prior to study commencement?	5 & 6
Participant knowledge of the interviewer	7	What did the participants know about the researcher? e.g. personal goals, reasons for doing the research	5 and title pag
Interviewer characteristics	8	What characteristics were reported about the inter viewer/facilitator? e.g. Bias, assumptions, reasons and interests in the research topic	Title page
**Domain 2: Study design**			
Theoretical framework			
Methodological orientation and Theory	9	What methodological orientation was stated to underpin the study? e.g. grounded theory, discourse analysis, ethnography, phenomenology, content analysis	6
Participant selection			
Sampling	10	How were participants selected? e.g. purposive, convenience, consecutive, snowball	5
Method of approach	11	How were participants approached? e.g. face-to-face, telephone, mail, email	5
Sample size	12	How many participants were in the study?	5 & 8
Non-participation	13	How many people refused to participate or dropped out? Reasons?	5 & 8
Setting			
Setting of data collection	14	Where was the data collected? e.g. home, clinic, workplace	5
Presence of non-participants	15	Was anyone else present besides the participants and researchers?	5
Description of sample	16	What are the important characteristics of the sample? e.g. demographic data, date	8
Data collection			
Interview guide	17	Were questions, prompts, guides provided by the authors? Was it pilot tested?	5
Repeat interviews	18	Were repeat inter views carried out? If yes, how many?	N/A
Audio/visual recording	19	Did the research use audio or visual recording to collect the data?	5
Field notes	20	Were field notes made during and/or after the inter view or focus group?	5
Duration	21	What was the duration of the inter views or focus group?	5
Data saturation	22	Was data saturation discussed?	N/A
Transcripts returned	23	Were transcripts returned to participants for comment and/or	6

Topic	Item No.	Guide Questions/Description	Reported on Page No.
		correction?	
**Domain 3: analysis and findings**			
Data analysis			
Number of data coders	24	How many data coders coded the data?	6
Description of the coding tree	25	Did authors provide a description of the coding tree?	6
Derivation of themes	26	Were themes identified in advance or derived from the data?	6
Software	27	What software, if applicable, was used to manage the data?	6
Participant checking	28	Did participants provide feedback on the findings?	No
Reporting			
Quotations presented	29	Were participant quotations presented to illustrate the themes/findings? Was each quotation identified? e.g. participant number	Yes
Data and findings consistent	30	Was there consistency between the data presented and the findings?	Yes
Clarity of major themes	31	Were major themes clearly presented in the findings?	Yes
Clarity of minor themes	32	Is there a description of diverse cases or discussion of minor themes?	Yes

Developed from: Tong A, Sainsbury P, Craig J. Consolidated criteria for reporting qualitative research (COREQ): a 32-item checklist for interviews and focus groups. *International Journal for Quality in Health Care*. 2007. Volume 19, Number 6: pp. 349 – 357

Once you have completed this checklist, please save a copy and upload it as part of your submission. DO NOT include this checklist as part of the main manuscript document. It must be uploaded as a separate file.
